# Multimodal Magnetic Resonance Imaging and Therapeutic Intervention With Yi-nao-jie-yu Decoction in a Rat Model of Post-stroke Depression

**DOI:** 10.3389/fpsyt.2020.557423

**Published:** 2020-11-04

**Authors:** Zijun Zhao, Wen Zhang, Yuan Zhang, Yun Zhao, Chunxiang Zheng, Huiling Tian, Jianfeng Lei, Yan Liu, Ruizhen Zhao, Qisheng Tang

**Affiliations:** ^1^Beijing University of Chinese Medicine, Beijing, China; ^2^Department of Pediatrics, Third Affiliated Hospital, Beijing University of Chinese Medicine, Beijing, China; ^3^Department of Neurology, Beijing Hospital of Traditional Chinese Medicine Shunyi Branch, Beijing, China; ^4^Department of Cardiology, Third Affiliated Hospital, Beijing University of Chinese Medicine, Beijing, China; ^5^Department of Encephalopathy, Third Affiliated Hospital, Beijing University of Chinese Medicine, Beijing, China; ^6^School of Acupuncture-Moxibustion and Tuina, Beijing University of Chinese Medicine, Beijing, China; ^7^Center for Medical Experiments and Testing, Capital Medical University, Beijing, China; ^8^School of Traditional Chinese Medicine, Beijing University of Chinese Medicine, Beijing, China; ^9^Center of Treating Potential Diseases, Third Affiliated Hospital, Beijing University of Chinese Medicine, Beijing, China

**Keywords:** post-stroke depression, diffusion tensor imaging, arterial spin labeling, magnetic resonance spectroscopy, yi-nao-jie-yu decoction, traditional Chinese medicine

## Abstract

Post-stroke depression (PSD) is the most common neuropsychiatric complication after a stroke, though its neuropathological characteristics have not been fully elucidated. Comprehensive and non-invasive magnetic resonance (MR) assessment techniques are urgently needed for current research, as diffusion tensor imaging (DTI), arterial spin labeling (ASL), and magnetic resonance spectroscopy (MRS) can allow for a comprehensive assessment of neuropathological changes in the brain. These techniques can provide information about microscopic tissue integrity, cerebral perfusion, and cerebral metabolism, and can serve as powerful tools for investigating neurophysiological changes associated with PSD. Yi-nao-jie-yu decoction (YNJYD) is a Chinese herbal formulation based on the theory of traditional Chinese medicine, with demonstrated clinical efficacy in the treatment of PSD. The aim of this study was to use these MR techniques to evaluate changes in PSD and YNJYD-treated rats. This is the first experimental study in animals to investigate neuropathological changes associated with PSD using a combination of multiple MR techniques, including DTI, ASL, and MRS. In addition, we investigated the effect of YNJYD in a rat model of PSD by assessing changes in brain tissue microstructure, brain metabolism, and cerebral perfusion. First, depressive-like behaviors of PSD rats were assessed by the open field test (OFT), sucrose preference test (SPT), and Morris water maze (MWM) test, and then the integrity of the rats' microstructure was assessed by DTI, the levels of regional cerebral perfusion were assessed by ASL, and changes in the relative concentrations of brain metabolites were determined by MRS. The results showed that OFT and SPT scores were significantly reduced in PSD rats, as was performance in the MWM; these PSD-associated changes were attenuated in rats administered YNJYD, with improved depressive-like behaviors evidenced by increased OFT and SPT scores and improved performance in the MWM task. Furthermore, we found that PSD rats had lower perfusion levels in the prefrontal cortex (PFC) and hippocampus (HP), microstructural damage, and abnormal changes in the concentrations of brain metabolites; YNJYD exerted therapeutic effects on PSD rats by improving microcirculation in the PFC and HP, regulating glutamatergic systems and membrane phospholipid metabolism, and repairing microstructural damage.

## Introduction

Stroke patients are often affected by psychological distress and neuropsychiatric disorders (1), and post-stroke depression (PSD) is the most common neuropsychiatric complication after a stroke. PSD can be disabling and can have a negative impact on recovery by reducing patients' quality of life and increasing the burden on caregivers. Approximately one-third of stroke patients suffer from PSD (2), resulting in a serious social and public health problem; thus, there is an urgent need to characterize the pathophysiological changes associated with PSD, its pathogenesis, and therapeutic regimens to treat it (3).

Having a complex pathophysiological mechanism, PSD is associated with several psychosocial factors and is closely related to the type of neurobiological dysfunction caused by ischemic injuries, such as those following a stroke; however, its pathogenesis and pathological characteristics have yet to be fully explored. Post-stroke psychiatric disorders are extrinsic manifestations of a range of pathological lesions, primarily involving the central nervous system. They may be accompanied by neuropathological abnormalities, such as changes in cerebral metabolism, cerebral blood flow (CBF), and cerebral microstructure, and comprehensive and non-invasive magnetic resonance (MR) assessments are urgently needed to better understand these changes. With the rapid development of MR technology, multimodal MR has become an important research tool in the field of neuropsychiatric disorders, but PSD-related MR studies are still very limited, and most have only employed a single detection method (4, 5). The pathological changes associated with neuropsychiatric disorders involve many factors, and a single detection method may not comprehensively reflect the abnormal changes occurring in the brain. Some studies have placed greater emphasis on metabolic cerebellar changes than on cerebral changes (6, 7), and the pathophysiological alterations occurring in key regions of the brain need to be investigated more comprehensively. Thus, for the first time, we aimed to combine the use of three MR techniques, diffusion tensor imaging (DTI), arterial spin labeling (ASL), and magnetic resonance spectroscopy (MRS) to assess changes in the brains of rats using a model of PSD. We performed a comprehensive assessment of the pathological characteristics of PSD, focusing on characterizing changes in microstructural integrity, cerebral perfusion, and brain metabolite levels, and we simultaneously assessed the neuroprotective potential of treating with a yi-nao-jie-yu decoction (YNJYD), a traditional Chinese medicine (TCM) for treating PSD, through these neuroimaging techniques. This investigation will provide useful clues for understanding the pathogenesis of PSD and for assessing therapeutic regimens to treat it.

In TCM, the therapeutic regimen for PSD treatment differs from those employed by Western medicine. Traditional Chinese medicine exerts therapeutic effects by enhancing the body's internal defense mechanisms and by regulating the functional balance of the human body; the use of YNJYD was established based on this theory. In preliminary studies, we found that the expression levels of brain-derived neurotrophic factor (BDNF) and vascular endothelial growth factor (VEGF) were reduced in the hippocampus (HP), hypothalamus, and amygdala, all regions comprising the Papez neural circuitry in rat models of PSD (8). Light microscopy revealed that the number of pyramidal cells in the hippocampal CA1 region was reduced in PSD rats; neuronal cell bodies shrank by varying degrees; the nuclei became condensed, fragmented, and dissolved; and cells were arranged in an irregular manner (9). After treatment with YNJYD, BDNF and VEGF concentrations became significantly elevated, and the morphological changes induced by PSD in the hippocampal CA1 region also significantly improved. The Papez circuit is a classical emotional regulatory circuit, forming the morphological basis of affective behavior. It is functionally important for memory storage and emotional stabilization and is regulated by the prefrontal cortex (PFC). The HP is a key component of the Papez circuit, and we have found, in previous studies, that various secondary depressive disorders, such as post-cerebral hemorrhage depression, post-cerebral ischemia depression, and post-partum depression, are associated with abnormalities in hippocampal neurotransmitters, phosphatidylinositol-related signaling pathways, as well as morphological changes in these structures (10–12). The PFC is recognized as one of the key sites of emotional regulation, and, as a higher “control center,” it is involved in the emotional regulation of the Papez neural circuitry. We have also previously found abnormalities in neurotransmitters and brain metabolism in the PFC in patients with neurological and psychiatric disorders (13, 14). Therefore, based on the importance of the HP and PFC in emotional regulation, we mainly focused on assessing changes in these two brain regions in a rat model of PSD and following treatment with YNJYD using MR techniques and behavioral testing to quantify changes in depressive symptoms.

## Materials and Methods

### Animals

The experimental procedures described in this study conformed to the ARRIVE guidelines and those described in the Guide for the Care and Use of Laboratory Animals issued by the National Institutes of Health and were approved by the Ethics Committee of Beijing University of Chinese Medicine. Specific-pathogen-free (SPF) male Wistar rats, aged 6 weeks and weighing 200 ± 10 g, were acquired from Beijing Vital River Laboratory Animal Technology Co., Ltd. [animal license: SCXK (Jing) 2016-0006], and were housed in the animal laboratory of Beijing University of Chinese Medicine. The room temperature was maintained at 22 ± 2°C, with a relative humidity of 50–60%, under a 12/12-h light/dark cycle (lights on at 8:00 a.m., lights off at 8:00 p.m.). The experimental animals were randomly divided into the following four groups (*N* = 6/group): a normal group, a PSD group, a YNJYD-treated group, and a sham-operated group.

### Model Preparation

#### Ischemic Stroke

The middle cerebral artery occlusion (MCAO) model was generated with reference to the intraluminal filament-based model described by Kuge et al. (15). The right common carotid artery (CCA), external carotid artery (ECA), and internal carotid artery (ICA) were isolated after the experimental animals were intraperitoneally anesthetized with sodium pentobarbital (50 mg/kg), and the proximal end of the CCA and the entire ECA were temporarily occluded with an arterial clamp. A small incision was made at the distal end of the CCA, followed by the insertion of a 4-0 nylon thread (Beijing Bioway Biological Co., Ltd., item No. 2838-A4) from the incision site into the ICA; the thread was slowly extended upward, into the origin of the middle cerebral artery (MCA). The rats in the sham-operated group received the same surgical procedure, but the MCA was not occluded. Two hours later, the filaments were removed, resulting in reperfusion injury as blood flow was restored.

#### Post-stroke Depression

**Figure 1:**
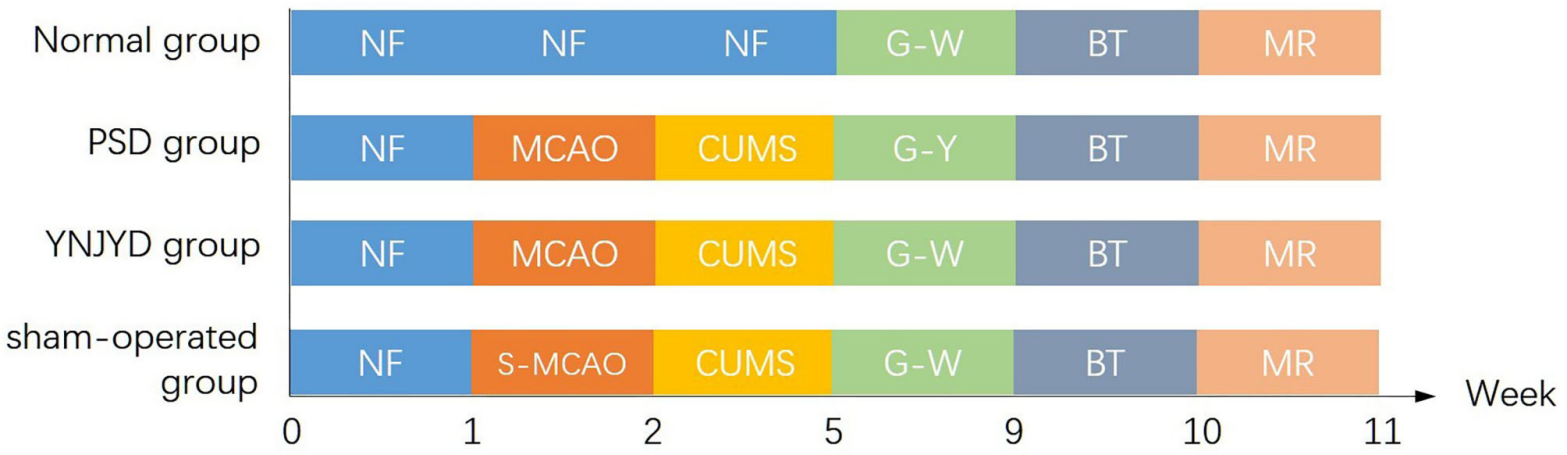
Experimental timeline of the four groups, including a normal group, a post-stroke depression (PSD) group, a yi-nao-jie-yu decoction (YNJYD)-treated group, and a sham-operated group, who underwent normal feeding (NF), middle cerebral artery occlusion operation (MCAO), sham operation for middle cerebral artery occlusion (S-MCAO), chronic unpredictable mild stress (CUMS), gavage-administered YNJYD (G-Y), gavage-administered distilled water (G-W), behavioral testing (BT), and magnetic resonance (MR) detection.

The PSD model employed isolation housing in combination with a chronic unpredictable mild stress (CUMS) paradigm following the MCAO operation. The CUMS procedure involved the use of seven different stressors, which were randomly applied each week, over a total of 3 weeks. The stressors were as follows: (1) fasting for 24 h; (2) water deprivation for 24 h; (3) 45° cage tilting, lasting for 24 h; (4) tail clamping for 5 min; (5) exposure to a wet cage for 24 h; (6) swimming in cold water (4°C) for 5 min; and (7) restricted movement for 4 h. In the sham-operated group, the CUMS protocol, combined with isolation housing, was carried out on the MCAO sham-operated rats. A schematic representation of the experimental design is depicted in Figure 1. All animals received the same behavioral testing and functional MRI (fMRI) detection. The “normal” group rats received no surgery and were not exposed to the CUMS paradigm.

### Drug Administration

YNJYD was provided by The Third Affiliated Hospital of Beijing University of Traditional Chinese Medicine. The quality of the YNJYD was controlled as outlined by the guidelines for granules in the 2010 edition of the Pharmacopeia. YNJYD mainly contains two kinds of Chinese herbal medicine: 20 g of *Acanthopanax senticosus* and 10 g of *Schisandra chinensis*. The granules were dissolved in 100 ml of distilled water and maintained at 4°C until further use. In our experiments, the drug dose selected for rats was converted based on the human and animal equivalent dose formula; YNJYD was administered by gavage once a day for 4 weeks. Rats in the YNJYD group were treated with YNJYD at a dose of 0.4 g/kg of body weight, while the normal group, PSD group, and sham group were administered an equivalent volume of normal saline based on body weight.

### Behavioral Tests

The behavioral tests were performed and scored by three trained and experienced observers who were blinded to the treatment/surgical conditions. All animals were subjected to behavioral testing at the end of gavage dosing.

#### Sucrose Preference Test (SPT)

All animals were food-deprived for 24 h before the test. Afterwards, two bottles containing either 1% sucrose solution (w/v; sucrose dissolved in distilled water) or distilled water were placed in the cage for rats to consume on test day. After 1 h, the consumption volume of each solution was recorded and analyzed based on the formula: percentage sucrose preference = (volume of sucrose solution consumption/total fluid intake volume) × 100.

#### Morris Water Maze (MWM)

MWM testing was conducted in a round, black pool 180 cm in diameter and 31 cm in depth. Pool temperature was maintained at 22 ± 1°C. The escape platform was placed in the center of one quadrant of the pool, submerged 1 cm beneath the water surface. The platform remained in the same position throughout the learning trials and was removed from the pool during the spatial probe test. Surrounding objects remained in the same position throughout the training and testing periods. The positional navigation experiment was carried out for 4 days, and each rat was tested four times a day. One rat was randomly selected to be placed into the water in each quadrant (facing the wall of the tank). Each animal could only be used once a day in each quadrant. The cutoff time limit to find the platform was 60 s. The time needed to find and climb onto the platform (escape latency) was recorded for each rat. The animals were allowed to rest on the platform for 30 s before starting the next round of training, facing in a new direction. If the hidden platform was not found within 60 s, the rats were guided to the platform by the experimenter, and these rats were also trained facing in the next direction after resting on the platform for 30 s. To examine changes in spatial memory, a spatial probe trial was performed 24 h after the last training session, during which the platform was removed from the pool and the rat was allowed to swim freely for 1 min. The escape latency and number of times the rats crossed the platform were recorded for comparisons between groups.

#### Open Field Test (OFT)

The inner surface of the OFT apparatus was painted black. The floor of the apparatus (100 × 100 × 50 cm) was divided into a grid of 25 identical squares (20 × 20 cm) with white stripes. In a dark, quiet room, a single rat was placed at the center of the arena and was allowed to explore freely for 3 min. The number of squares crossed by the rats (defined as three or four paws crossing the line, or more than half of the body of the rat entering an adjacent square; each line crossed was scored as one point) and the frequencies of rearing behaviors (each rearing event was scored as one point) were quantified. The total OFT score was determined as the sum of these two measures. After each test, the apparatus was thoroughly cleaned with 75% ethanol.

### MR Data Acquisition

MR detection was performed using a 7.0-T MRI scanner (Bruker, Germany) in the small animal *in vivo* imaging room of the Experimental Center of Capital Medical University. Before the MRI, anesthesia was induced with a mixture of 5% isoflurane and 95% oxygen in a gas anesthesia apparatus (Phoenix, Germany), and anesthesia was maintained with a gas mixture of 2% isoflurane and 98% O_2_, with the rat placed in the center of the magnetic field. The body temperature of the animals was carefully maintained at a normal, physiological level, and respiratory rate was closely monitored using a Small Animal Monitoring and Gating System (SA Instruments, Inc. Stony Brook, NY, USA). T2-weighted images were first collected using a spin-echo sequence with the following parameters: TR (repetition time) = 5,100 ms, TE (echo time) = 33 ms, MTX (matrix size) = 256 × 256, SR (spatial resolution) = 0.136 mm/pixel, ST (slice thickness) = 1 mm, 27 slices in total, and FOV (field of view) = 3.5 × 3.5 cm. The following sequence of MR techniques was used to measure parameters, coronally.

#### DTI

A single-shot spin-echo plane was used to measure the fractional anisotropy (FA) and apparent diffusion coefficient (ADC). TR = 6,752 ms, TE = 22 ms, FOV = 3.5 × 3.5, MTX = 128 × 128, SR = 0.273 mm/pixel, NEX (number of excitations) = 1, ST = 1 mm; the diffusion gradient encoding direction was 30, 2*b* = 0 and 1,000 s/mm^2^ (where *b* = diffusion weighting coefficient). The scan time was 17 min.

#### ASL

The echo-plane Flow Sensitive Alternating Inversion Recovery (FAIR) technique was used to measure CBF values. TR = 18,000, TE = 25 ms, FOV = 3.0 × 3.0 cm, MTX = 128 × 128, NEX = 1, ST = 2.00 mm, and TIR (triple inversion recovery) value = 22.

#### MRS

After shimming and water suppression, ^1^H spectra were acquired using a PRESS (Point RESolved Spectroscopy) sequence with 2018 sampling points, TR = 2,500 ms, TE = 20 ms, FOV = 3 × 2 × 2 (for PFC), FOV = 3 × 3 × 2 (for HP), and MTX = 256 × 256, and the resulting spectra were Fourier transformed; after the baseline correction, the spectra were measured at the corresponding chemical shifts.

**Figure 2:**
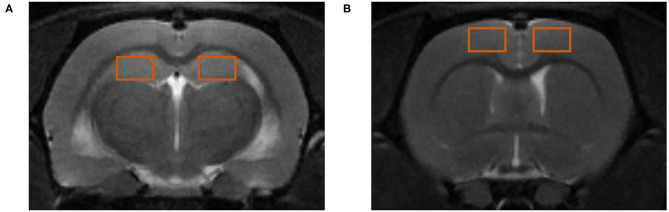
Regions of interest (ROIs) for magnetic resonance (MR) detection. **(A)** ROIs in the hippocampus (HP). **(B)** ROIs in the prefrontal cortex (PFC).

Both the PFC and HP were selected as regions of interest (ROIs), as shown in Figure 2. DTI and ASL images were processed using ParaVision version 5.1 software (Bruker BioSpin Corporation, MA, USA). After the importation of MRS data into Paravision, configured TopSpin 2.0 software (Bruker BioSpin Corporation, MA, USA) was used to obtain the metabolite spectra, the baseline correction was performed to calculate the area under the curve, and the NAA/Cr (N-acetyl aspartate/creatine), Cho/Cr (choline/creatine), Glu/Cr (glutamate/creatine), and mI/Cr (myo-inositol/creatine) ratios were determined for comparisons between groups.

### Statistical Analyses

All data were processed and analyzed using Statistical Package for the Social Sciences version 20.0 (IBM Corporation, Armonk, NY, USA). The experimental data were all normally distributed, as determined by the Shapiro-Wilk test, and described using means and standard deviations (x̄± S). After assessing the homogeneity of variance in each group, one-way analysis of variance (ANOVA) was used to compare differences between groups, and Fisher's Least Significant Difference test was used for intergroup pairwise comparisons. *P*-values < 0.05 were considered statistically significant.

## Results

### Effect of YNJYD Administration on Depressive-Like Behavior

**Figure 3:**
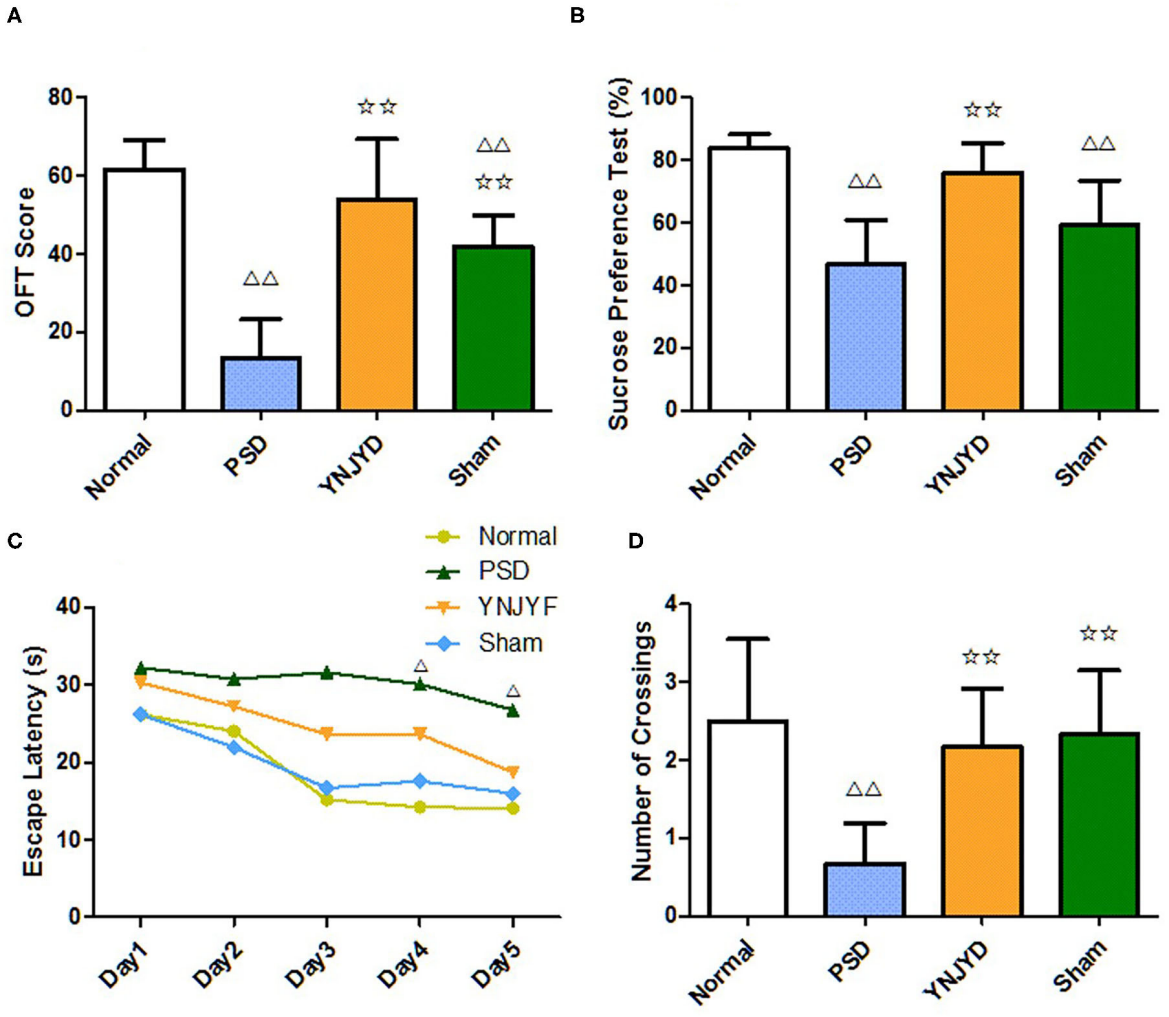
Effects of yi-nao-jie-yu decoction (YNJYD) on depressive-like behaviors in normal and post-stroke depression (PSD) model rats. **(A)** The open field test (OFT) scores, quantified from the sum of the crossing times and rearing times. **(B)** Percentages for the sucrose preference testing (SPT). **(C)** Escape latency in the Morris water maze (MWM) test. **(D)** The number of platform crossings in the MWM test. The data are presented as means ± SD. ^Δ^*P* < 0.05 vs. normal; ^ΔΔ^*P* < 0.01 vs. normal; ✩✩*P* < 0.01 vs. PSD.

We conducted a series of behavioral tests, including the OFT, SPT, and MWM, to examine the effect of YNJYD administration on depressive-like behaviors in normal and PSD rats. In our study, there was a lot of evidence to suggest that YNJYD administration could relieve depressive-like behaviors in rats. First, as shown in the OFT results presented in Figure 3A, the OFT scores were significantly lower in the PSD group than in the normal group (*P* < 0.01); the YNJYD-treated group had higher OFT scores than the untreated PSD group (*P* < 0.05), but did not differ significantly from the normal group (*P* > 0.05). Second, in the SPT test, rats in the PSD group had a significantly lower sucrose preference than those in the normal group (*P* < 0.01), and sucrose consumption was significantly higher in rats treated with YNJYD than those in the PSD group that were untreated (Figure 3B). In addition, in the MWM, we found that the PSD group had significantly fewer platform crossings than the other groups in the probe test (*P* < 0.01); however, the number of crossings in the YNJYD-treated group was not statistically different from the number of crossings in the normal group (Figure 3D). As shown in Figure 3C, in the location–navigation testing on days 1–4 and the probe test on day 5, there were no significant differences in escape latency across the groups during the first 3 days; the latency significantly increased in the PSD group on days 4–5 (*P* < 0.05), though the YNJYD-treated group did not significantly differ from the normal group or the sham-operated group (*P* > 0.05). The test results in the sham-operated group were similarly noteworthy. The OFT scores (*P* < 0.01; Figure 3A) and SPT measures (*P* < 0.05; Figure 3B) in the sham-operated group were significantly lower than the normal group, while in the MWM, the number of platform crossings did not statistically differ from the normal group (*P* > 0.05; Figure 3D).

### Effect of YNJYD on PSD-Induced Changes in Microstructural Integrity Assessed by DTI

**Figure 4:**
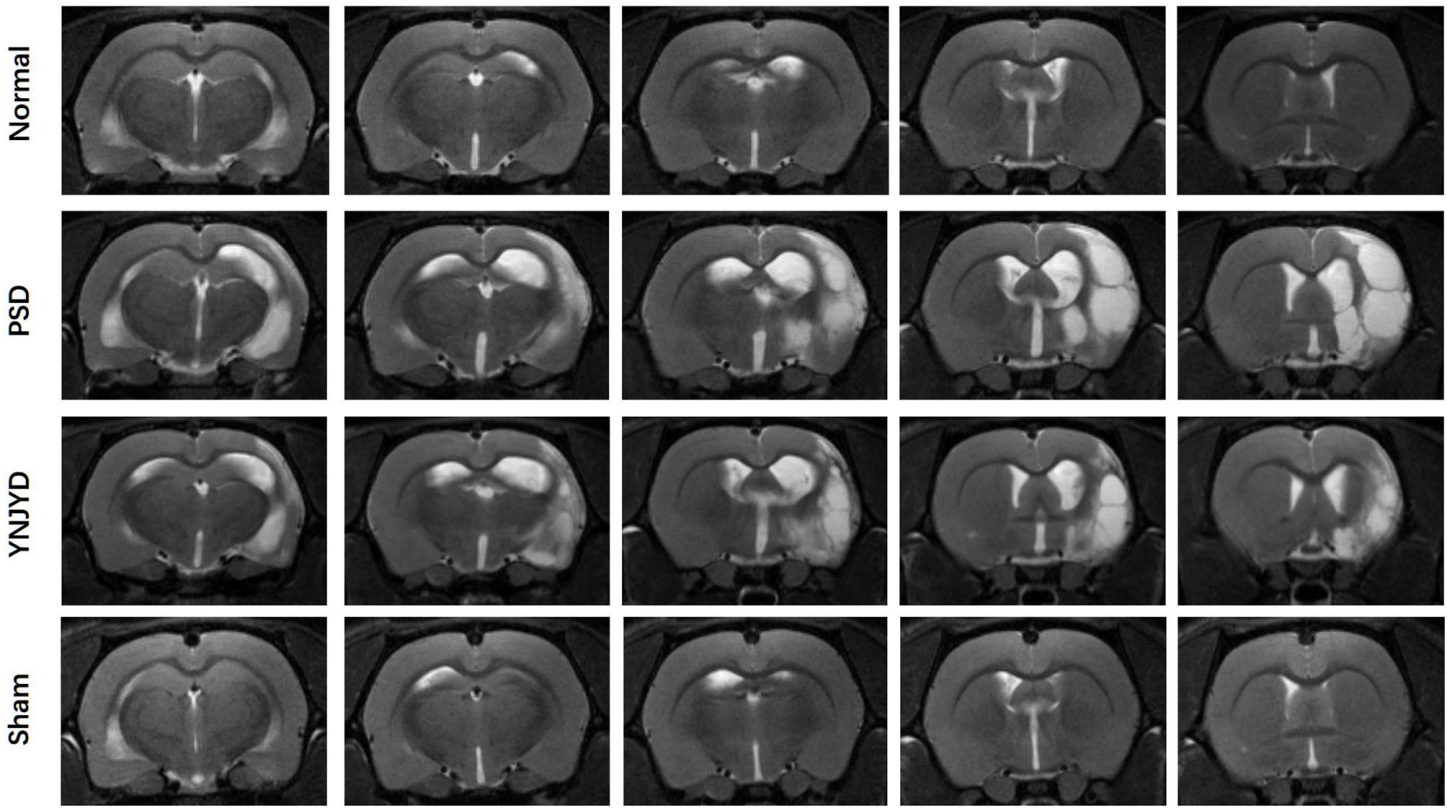
T2-weighted magnetic resonance imaging of normal, post-stroke depression (PSD), YNJYD-treated, and sham-operated rats.

**Figure 5:**
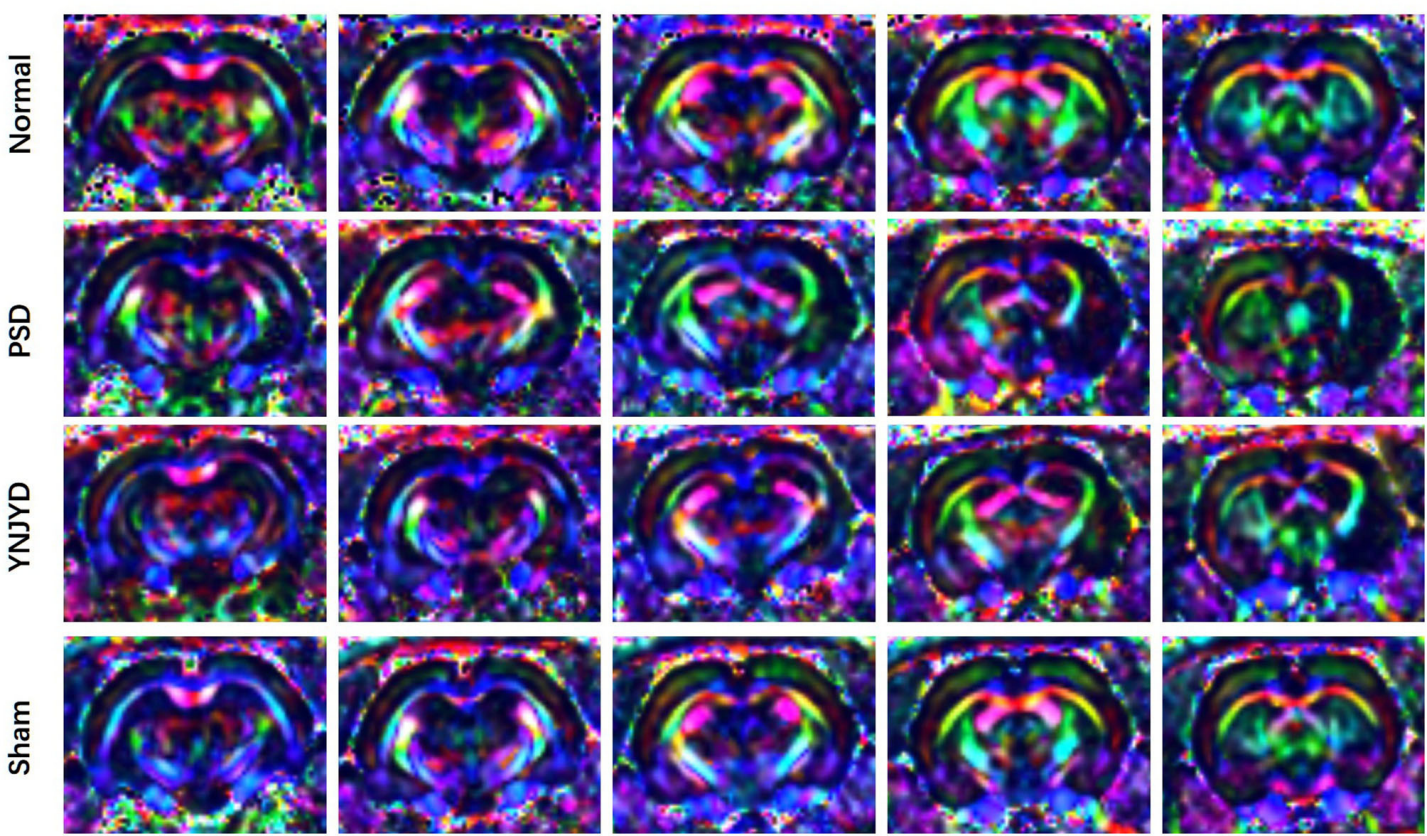
Representative color-encoded fractional anisotropy (FA) directional maps from continuous scanning in normal, post-stroke depression (PSD), YNJYD-treated, and sham-operated rats.

**Figure 6:**
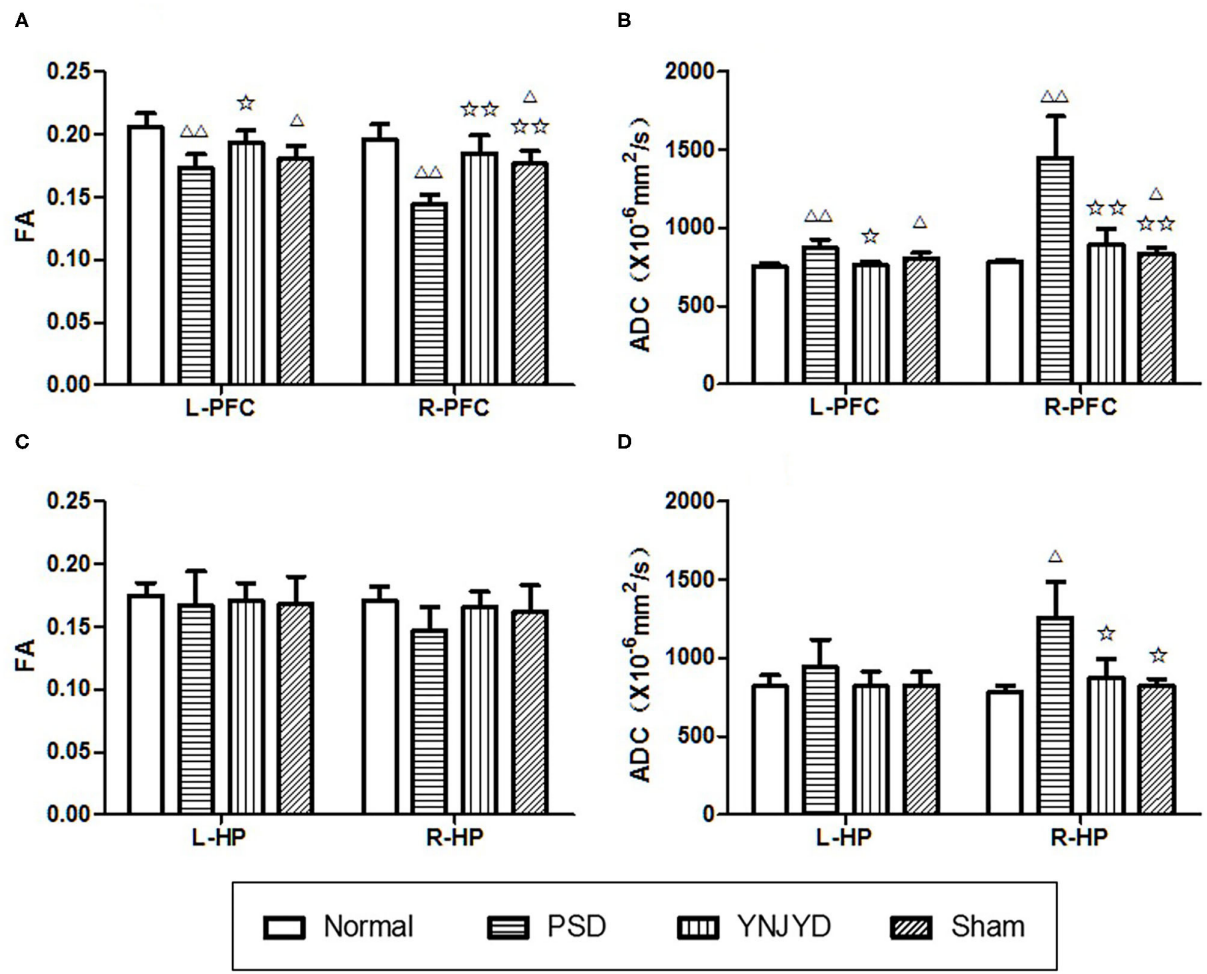
Effects of yi-nao-jie-yu decoction (YNJYD) administration on changes in fractional anisotropy (FA) and apparent diffusion coefficient (ADC) levels. **(A)** FA level in the bilateral prefrontal cortex (PFC). **(B)** ADC level in the bilateral PFC. **(C)** FA level in the bilateral hippocampus (HP). **(D)** ADC level in the bilateral HP. L = left side; R = right side. The data are presented as means ± SD. ^Δ^*P* < 0.05, ^ΔΔ^*P* < 0.01 vs. normal; ✩*P* < 0.05, ✩✩*P* < 0.01 vs. PSD (post-stroke depression) rats.

We first performed a T2-weighted MR scan of each rat, as shown in Figure 4, and then we quantitatively analyzed the microstructural integrity of the PFC and HP by measuring the FA and ADC values of the ROIs. Figure 5 shows the far-field pattern of consecutive scans of normal and PSD rats treated with YNJYD, and the sham-operated group. As shown in Figures 6A,B, in the PFC, the PSD rats had significantly lower FA values and higher ADC values (*P* < 0.01), respectively, compared to normal rats; however, FA and ADC values nearly returned to normal baseline levels following treatment with YNJYD. A decrease in FA values and an increase in ADC values was also observed in the sham-operated group (*P* < 0.05), but the magnitude of change from the normal group was not as large as its difference compared to the PSD group. In the bilateral HP assessment shown in Figures 6C,D, the ADC value of the right HP was significantly elevated relative to the PSD group rats (*P* < 0.05), and the ADC value in the YNJYD-treated group was lower than that in the PSD group (*P* < 0.05) but did not significantly differ from that of the normal group (*P* > 0.05; Figure 6D). In addition, the rats in the PSD group had relatively higher ADC values in the left HP, but this difference was not statistically significant (*P* > 0.05).

### Effect of YNJYD Administration on Cerebral Perfusion Assessed by ASL

**Figure 7:**
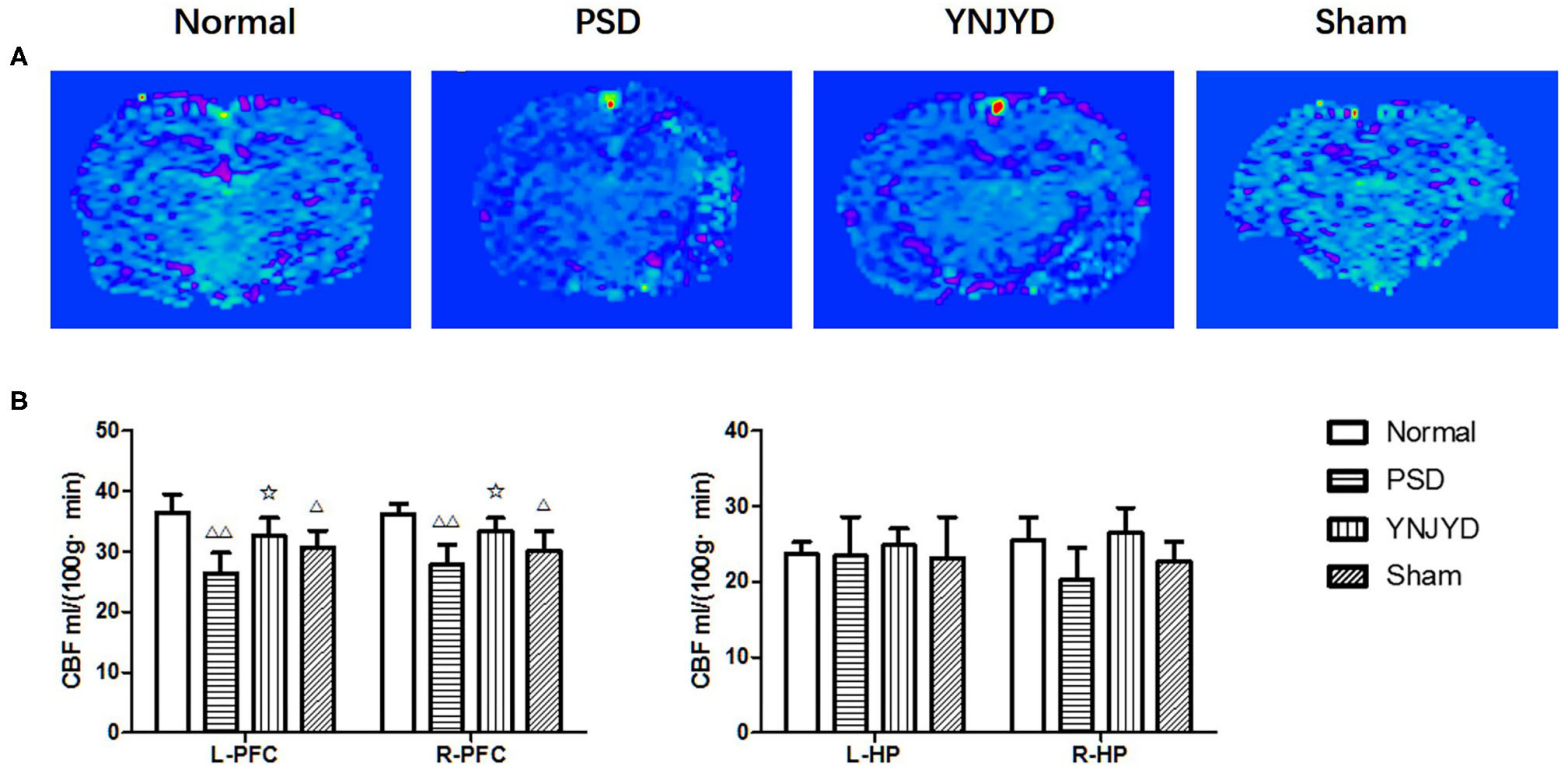
Effects of yi-nao-jie-yu (YNJYD) administration on changes in cerebral blood flow (CBF) detected by arterial spin labeling (ASL) in normal, post-stroke depression (PSD), YNJYD-treated, and sham-operated rats. **(A)** ASL magnetic resonance (MR) images of the four groups. **(B)** Analysis of CBF in the left (L) and right (R) prefrontal cortex (PFC) and hippocampus (HP). The data are presented as means ± SD. ^Δ^*P* < 0.05, ^ΔΔ^*P* < 0.01 vs. normal; ✩*P* < 0.05.

MR ASL images are presented in Figure 7A. We found that the rats in the PSD group exhibited statistically significant hypoperfusion in the bilateral PFC (*P* < 0.01) compared to normal control rats, as shown in Figure 7B (left panel). There was no statistically significant difference in perfusion levels between normal animals and those treated with YNJYD. In addition, the sham-operated group also exhibited lower perfusion levels in the bilateral cortical regions (*P* < 0.05) compared to the normal group. However, in the bilateral HP, we did not find significant differences in perfusion levels across groups (*P* > 0.05), as shown in Figure 7B (right panel).

### Effect of YNJYD Decoction Administration on the Relative Concentrations of Brain Metabolites Assessed by MRS

**Figure 8:**
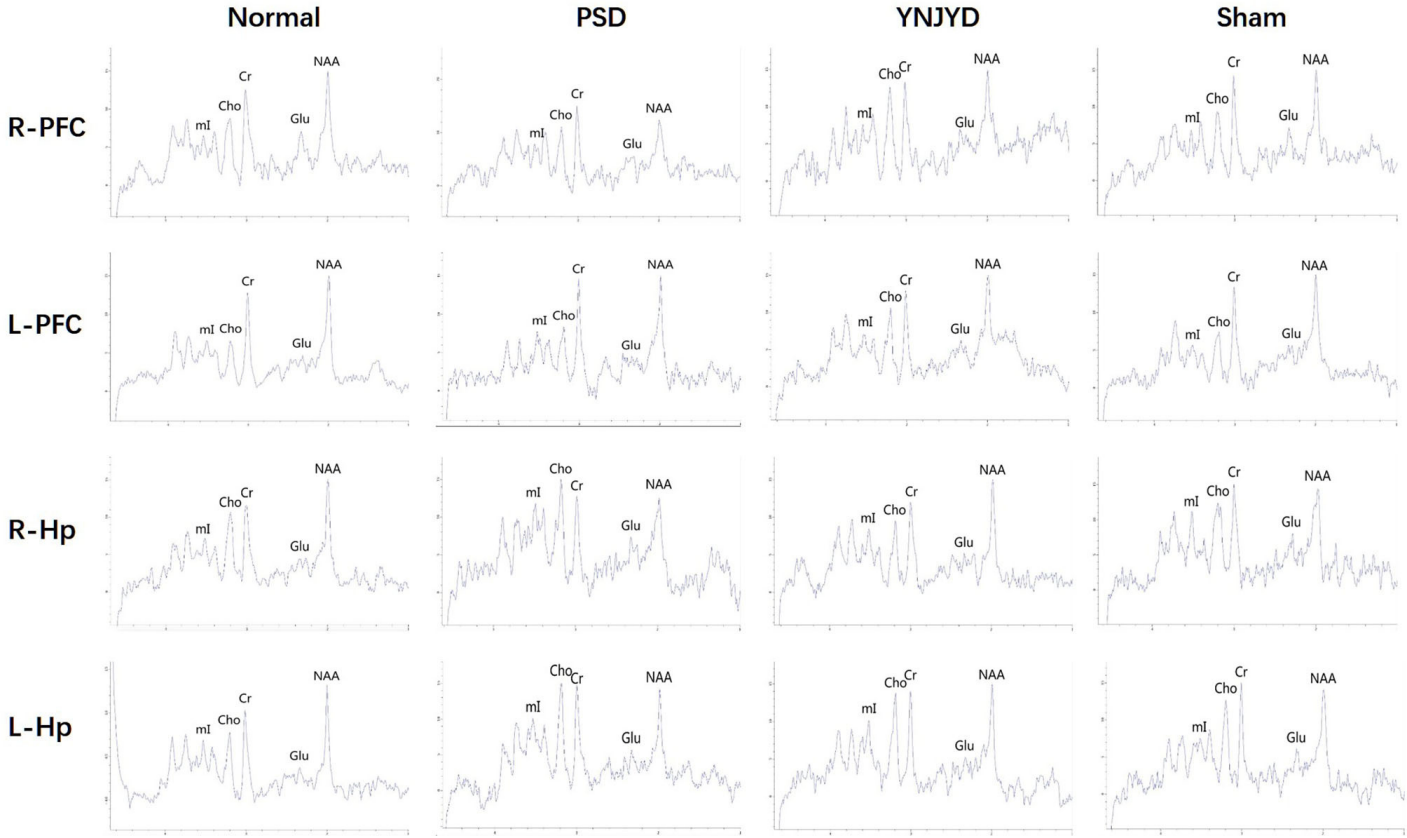
Representative magnetic resonance spectroscopy (MRS) spectra from the right (R) and left (L) prefrontal cortex (PFC) and hippocampus (HP) of normal, post-stroke depression (PSD), YNJYD-treated, and sham-operated rats. ^1^H MRS exhibits a creatine (Cr) peak at 3 ppm, an N-acetyl aspartate (NAA) peak at 2 ppm, a choline (Cho) peak at 3.2 ppm, a glutamate (Glu) peak at 2.2 ppm, and a myo-inositol (mI) peak at 3.6 ppm. Quantification of the relative ratio of each metabolite to Cr in each of the regions of interest (ROIs).

**Figure 9:**
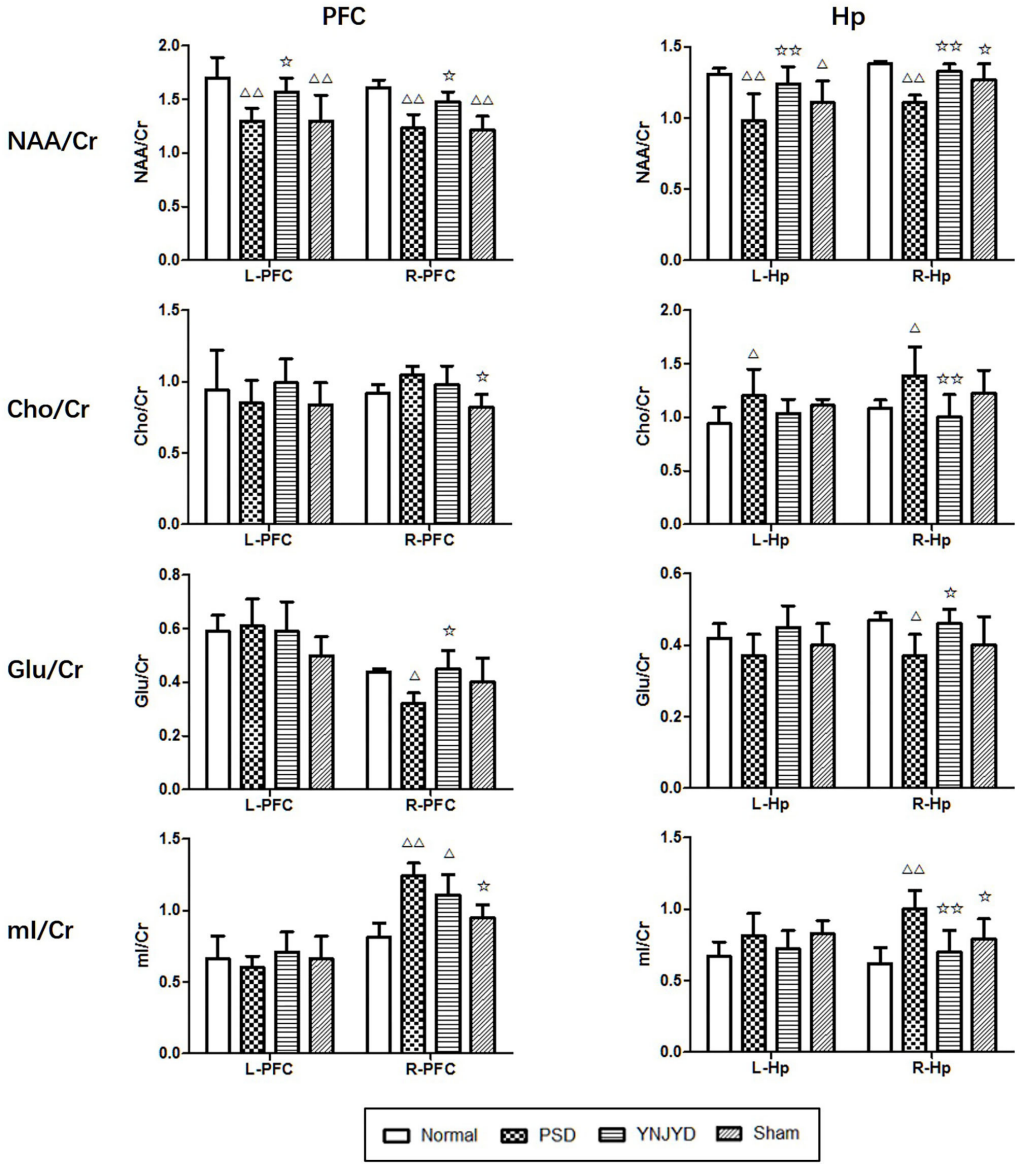
Effects of yi-nao-jie-yu decoction (YNJYD) and post-stroke depression (PSD) on the relative concentrations of brain metabolites, including N-acetyl aspartate (NAA), choline (Cho), glutamate (Glu), and myo-inositol (mI) ratios in the left (L) and right (R) side of the prefrontal cortex (PFC; left column), and the left and right side of the hippocampus (HP; right column) of normal, PSD, YNJYD-treated, and sham-operated rats. The data are presented as means ± SD. ^Δ^*P* < 0.05, ^ΔΔ^*P* < 0.01 vs. normal rats; ✩*P* < 0.05, ✩✩*P* < 0.01 vs. PSD rats.

Figure 8 shows representative MRS images of the bilateral HP and PFC of each of the four groups and Figure 9 shows the quantification of the NAA/Cr, Cho/Cr, Glu/Cr, and mI/Cr ratios in the bilateral PFC (left column) and HP (right). Compared to the normal group, the NAA/Cr ratio was significantly reduced in the bilateral HP and PFC of PSD rats (*P* < 0.01). The Glu/Cr ratio was also statistically significantly decreased in the right HP and PFC (*P* < 0.05), and significant increases were observed in the mI/Cr and Cho/Cr ratios in the right HP and PFC (*P* < 0.01), along with a significant elevation in the Cho/Cr ratio in the bilateral HP (*P* < 0.05). However, compared to the untreated PSD group, the above parameters recovered to varying extents in the YNJYD-treated group, and the mI/Cr ratio in the right PFC was still higher in the YNJYD group than in the normal group (*P* < 0.05). The other ratios measured in the YNJYD group did not significantly differ from those in the normal group (*P* > 0.05). In the left PFC and HP, no statistical differences were found in Glu/Cr or mI/Cr ratios across groups (*P* > 0.05). Notably, the sham-operated group exhibited a significant decrease in the NAA/Cr ratio in the bilateral PFC (*P* < 0.01), and there was also a statistically significant decrease in the NAA/Cr ratio in the left HP (*P* < 0.05).

## Discussion

The aim of the present study was to explore neuropathological changes associated with PSD and the underlying mechanisms by which YNJYD exerts antidepressant effects in PSD rats. We found that administration of YNJYD could significantly improve depressive-like behaviors in PSD rats. More importantly, PSD model rats exhibited distinct pathophysiological changes, including decreased local brain tissue perfusion levels, cerebral metabolic disorders, and fiber bundle injuries, which could be reversed by the administration of YNJYD. The present study showed that YNJYD could improve the PSD-induced changes in the HP and PFC of rats, including restoring decreased perfusion levels, regulating deficits in brain metabolism, and repairing loss of tissue integrity, leading to better behavioral performance in the testing paradigms.

We assessed the regional brain tissue blood flow by ASL. Arterial spin labeling is a completely non-invasive and quantifiable MR cerebral perfusion technique that can be performed without the need for exogenous contrast; regional CBF can be determined by measuring CBF values (16, 17), but there has been no study applying this technique to PSD model rats. As early as 1995, Spanish scholars (18) found a significant correlation between PSD and CBF hypoperfusion using single photon emission computed tomography, though there have been few cerebral perfusion studies on PSD since that time. In the present study, we found that the rats in both the PSD group and the sham-operated group had significantly reduced levels of cerebral perfusion in the bilateral PFC; the CBF values in the right PFC of PSD rats were statistically further reduced compared with the sham-operated group, while the cerebral perfusion levels of YNJYD-treated rats were significantly improved. In recent years, the results of cerebral perfusion studies on depression have been relatively consistent, and it is believed that depression is significantly associated with lower blood perfusion to certain brain regions (19, 20), with particular emphasis placed upon the abnormalities in hippocampal functional activity and blood perfusion in depressed patients (21–23). A number of studies assessing changes in cerebral perfusion in severely depressed patients have found that the tissues with CBF abnormalities are mostly concentrated in the frontal-limbic system (24, 25). However, other studies have reported no significant differences in CBF in these regions in patients with mild depression (26). In the present study, we did not detect significantly abnormal CBF values in the HP of PSD rats, which may suggest that PSD and primary depression may result from different pathophysiological mechanisms. In addition, the PSD rats in the sham-operated group did not exhibit a significant decrease in CBF values in the HP, which may be related to depression severity. We observed that although the sham-operated group exhibited a significant decrease in CBF values in the bilateral PFC, this change was smaller in magnitude than that observed in the PSD group, which might suggest that secondary brain injury caused by a stroke could aggravate the hypoperfusion seen in the PFC, and, to some extent, was more likely to lead to the emergence or aggravation of depressive symptoms. It is currently believed that the development of depression is associated with a disequilibrium between norepinephrine (NE) and 5-hydroxytryptamine (5-HT; serotonin) levels, as noradrenergic and serotonergic neurons are located in the brainstem, and their axons project to the frontal cortex through the thalamus and basal ganglia. The decrease in CBF in the PFC can affect the integrity and function of the serotonergic and noradrenergic neural pathways within this region, further resulting in a decrease in concentrations of 5-HT and NE and leading to the development of depressive symptoms. In the present study, the CBF in the bilateral PFC in the PSD rats treated with YNJYD was significantly higher than that of rats in the untreated PSD group, suggesting that YNJYD could be efficacious in treating PSD by improving cerebral perfusion in the PFC, leading to improved tissue repair mechanisms.

Cerebral blood flow provides the oxygen and glucose required to maintain neuronal function, and it is linked to neuronal activity through neurovascular coupling (27). Local hypoperfusion may be indicative of the functional decline of related neural tissues or damage to the tissue microstructure. Therefore, we further examined changes in brain tissue microstructure and metabolite concentrations in PSD rats. To be able to quantify the integrity of brain tissues, we employed the relatively new DTI technique, which mainly assesses microstructural changes in brain tissue by measuring the motility of water molecules (28, 29). In recent years, DTI has been increasingly used to study neuropsychiatric disorders, and FA and ADC are the most common measures of DTI.

FA can reflect the integrity of tissue microstructure and is considered to be an indirect, quantitative measure of nerve fiber density and degree of myelination. A decrease in the FA value is generally considered to be related to the pathological processes associated with structural trabecular destruction, fiber bundle damage, and myelin edema. The ADC value comprehensively describes microscopic movement of water molecules, reflecting the degree of diffusion. An increase in the ADC value may be indicative of enhanced diffusion of water molecules resulting from structural damage to the cell membrane and the subsequent expansion of the extracellular space. Currently, there are few DTI studies assessing changes associated with PSD, but several studies on patients with severe depression have shown that there are neural microstructural abnormalities, such as fiber tract damage and neuronal atrophy, in the HP and PFC (30–32). In the present study, assessment of hippocampal changes in PSD model rats only revealed elevated ADC values in the right HP compared to the normal group, while no significant change in the FA value of the right HP was observed. The reasons for this outcome are unknown but may be due to one or more of the following causes: (1) during the course of PSD, hippocampal neurons were damaged and the extracellular space became enlarged, without changing the structural features of the tissue, which would result in the ADC value changing significantly relative to the FA value; (2) the HP included both gray and white matter structures, and the white matter fiber bundles projected in different directions, resulting in a lower sensitivity of using the FA value to infer changes in the tissue microstructure of the HP. In our assessment of the PFC, we found that the FA value was significantly lower and the ADC value was significantly higher, bilaterally, in the PSD group than in the normal group, suggesting that there was microstructural damage in the PFC of PSD model rats. The FA values of the bilateral PFC and the ADC value of the left PFC in the sham-operated group were also significantly changed compared to the normal group, but the magnitude of change was smaller than that seen in the PSD group. Whether patients with primary depression also have microstructural damage to the PFC, however, is unknown and requires further study. In addition, we also observed that the FA value in the right PFC was significantly lower in the sham-operated group than in the normal group, while the ADC value did not differ; a possible explanation for this might be related to glial cell proliferation in this region, and migration/infiltration of glial cells into the enlarged extracellular space. Compared to the PSD group, the FA values of the bilateral PFC in the YNJYD group were significantly higher, while the ADC values were lower. These findings suggest that the bilateral PFC and the right HP of PSD rats exhibited structural abnormalities in nerve cells and fiber bundles and that YNJYD administration could protect and repair the damage to the brain tissue microstructures of regions such as the PFC and HP.

In previous studies, we found that PSD rats displayed changes in cerebral perfusion and the microscopic organization of tissue; detection of changes in CBF might be reflective of significant metabolic abnormalities. In this study, we assessed changes in concentrations of brain metabolites using ^1^H-MRS. We observed that the relative concentrations of NAA in the bilateral HP and the PFC of PSD rats were significantly reduced, suggesting neuronal loss and metabolic dysfunction, consistent with the findings of a number of previous MRS studies of depressive patients (33–35). In addition, we observed that the relative concentration of NAA in the bilateral PFC and the left HP of sham-operated rats was significantly decreased, while the NAA/Cr ratio in the right HP trended toward a decrease, but the differences were not statistically significant (*P* = 0.10). A significant negative correlation between bilateral hippocampal NAA/Cr ratios and Hamilton Depression Rating Scale scores has been found in a previous study (36). We observed that the concentration of NAA in the right HP of rats in the PSD group was significantly lower than that in the sham-operated group, which might suggest that the PSD was more pronounced than the primary depressive symptoms under the same stress conditions. In addition, we found an elevation in the relative concentrations of Cho in the bilateral HP of PSD rats, which might indicate that PSD rats had abnormal cell turnover and structural damage of lipids in the cell membrane, myelin sheath, and brain; PSD was also associated with brain injury, neurodegeneration, and proliferation of reactive stellate cells and astrocytes. Most studies have reported reduced glutamate levels in frontal regions of the brains of depressive patients compared to non-depressive populations (34, 37). However, there has been no uniform consensus about the degree of change in the concentration of Glu in the brain of PSD patients (38, 39). We observed a significant decrease in the Glu/Cr ratio in the right HP and PFC in the PSD group, suggesting a disturbance in the metabolic cycle regulating the compositions of the phospholipid membrane and glutamate metabolism that affected neuronal function in the HP and PFC. In addition, the mI/Cr ratio was elevated in the right HP and right PFC of PSD rats, suggesting that there may be a proliferation of glial cells and confirming that dysregulation of the second messenger system might play a role in the pathophysiological mechanisms of severe depression. In the present study, after administration of YNJYD to PSD rats, the relative concentrations of NAA and Glu were elevated, and the abnormally high relative concentrations of Cho and mI induced by PSD were attenuated, suggesting that YNJYD could effectively repair neurons after cerebral ischemia by promoting nervous system remodeling and by regulating the metabolic activity of the glutamatergic system and the phospholipid bilayer of the cell. Thus, YNJYD administration could play a therapeutic role in the treatment of PSD.

In TCM theory, the pathogenesis of PSD results from stagnation of the liver-qi and renal deficiency. Stagnation of the liver-qi can lead to blood stasis, which, combined with renal deficiency, may induce PSD. According to this theory, we formulated YNJYD, which has been shown to invigorate the kidneys, dispersing the depressed liver energy and normalizing qi-related functional activities. YNJYD is formulated strictly based on the compatibility theory of TCM, and its main pharmacological activity comes from compounds in two plants: *A. senticosus* and *S. chinensis*. *Acanthopanax senticosus* is the dried root and rhizome or leaf of the Araliaceae plant, and it functions by supplementing qi, strengthening the spleen, tonifying the kidneys, and tranquilizing the mind. A number of studies have reported that *A. senticosus* has obvious effects on the central nervous system, including neuroprotective, cognitive-enhancing, sleep-improving, antidepressant, and anxiolytic effects (40–45). *Schisandra chinensis* functions as an astringent, arrests discharge, invigorates qi, promotes the production of bodily fluids, and nourishes the kidneys and heart. Modern pharmacological studies have shown that the key neuroprotective mechanisms of *Schisandrae fructus* are a result of its anti-oxidative properties, its ability to inhibit apoptosis, and its anti-inflammatory and neurotransmitter-regulating mechanisms, which are important for treating a wide range of cardiovascular and cerebrovascular diseases, neurodegenerative diseases, as well as depression and other illnesses (46–48).

Although we evaluated PSD-induced changes in the brains of rats using multiple MRI techniques, the present study was still somewhat limited; we only performed a comparison within our rat model with YNJYD and did not have a positive control group. Therefore, our study confirmed the effectiveness of YNJYD in attenuating the functional, morphological, and associated behavioral changes induced by PSD, but there was no comparison to the effects induced by classical antidepressants, requiring further investigation. In addition, we have not yet assessed the serum YNJYD level in rats. Next, we will carry out serum pharmacology testing for YNJYD. During serum testing, we will consider the relationship between changes in serum-related indicators and MRI indicators to explore their correlation.

## Conclusions

In conclusion, this is the first study to examine the neuropathological changes in PSD model rats using multimodal MR techniques, and it is the first to use these techniques to assess the neuroprotective effects of TCM. First, through a series of behavioral tests, we confirmed that YNJYD could reduce depressive-like behaviors in rats by improving motor activity, by attenuating PSD-induced changes in sucrose preference, and by improving learning and memory abilities. Based on the lack of studies using multimodal MR techniques on PSD model animals, we explored the possible neuropathological changes characteristic of PSD and evaluated the neuroprotective effects of YNJYD. Consistent with the behavioral changes we observed, we also found that PSD rats had decreased PFC and HP perfusion, microstructural damage, and abnormal changes in brain metabolites. Administration of YNJYD has therapeutic potential to improve microcirculation in the PFC and HP, to regulate the metabolic activity that affects the glutamatergic system and membrane phospholipids, and to repair the microstructural changes associated with PSD to generate a positive emotional state. These findings provide a new perspective for TCM-related treatment of neuropsychiatric disorders following a stroke.

## Data Availability Statement

All datasets generated for this study are included in the article/supplementary material.

## Ethics Statement

The animal study was reviewed and approved by Ethics Committee of Beijing University of Chinese Medicine.

## Author Contributions

QT and RZ conceived the design of the project. ZZ, WZ, QT, and RZ contributed to writing and editing the manuscript. ZZ and WZ performed the data analysis. ZZ, YZhang, YZhao, and CZ performed the experiments. HT contributed to the preparation of the MCAO animal model. JL performed the magnetic resonance imaging. YL provided access to behavioral equipment and participated in behavioral testing. QT supervised the project. All authors have read and agreed to the publication of the manuscript.

## Conflict of Interest

The authors declare that the research was conducted in the absence of any commercial or financial relationships that could be construed as a potential conflict of interest.
